# Use of a Real-Time Training Software (Laerdal QCPR^®^) Compared to Instructor-Based Feedback for High-Quality Chest Compressions Acquisition in Secondary School Students: A Randomized Trial

**DOI:** 10.1371/journal.pone.0169591

**Published:** 2017-01-05

**Authors:** Andrea Cortegiani, Vincenzo Russotto, Francesca Montalto, Pasquale Iozzo, Roberta Meschis, Marinella Pugliesi, Dario Mariano, Vincenzo Benenati, Santi Maurizio Raineri, Cesare Gregoretti, Antonino Giarratano

**Affiliations:** Department of Biopathology and Medical Biotechnologies (DIBIMED), Section of Anesthesia Analgesia Intensive Care and Emergency, Policlinico P. Giaccone, University of Palermo, Palermo, Italy; Cardiff University, UNITED KINGDOM

## Abstract

High-quality chest compressions are pivotal to improve survival from cardiac arrest. Basic life support training of school students is an international priority. The aim of this trial was to assess the effectiveness of a real-time training software (Laerdal QCPR^®^) compared to a standard instructor-based feedback for chest compressions acquisition in secondary school students. After an interactive frontal lesson about basic life support and high quality chest compressions, 144 students were randomized to two types of chest compressions training: 1) using Laerdal QCPR^®^ (QCPR group– 72 students) for real-time feedback during chest compressions with the guide of an instructor who considered software data for students’ correction 2) based on standard instructor-based feedback (SF group– 72 students). Both groups had a minimum of a 2-minute chest compressions training session. Students were required to reach a minimum technical skill level before the evaluation. We evaluated all students at 7 days from the training with a 2-minute chest compressions session. The primary outcome was the compression score, which is an overall measure of chest compressions quality calculated by the software expressed as percentage. 125 students were present at the evaluation session (60 from QCPR group and 65 from SF group). Students in QCPR group had a significantly higher compression score (median 90%, IQR 81.9–96.0) compared to SF group (median 67%, IQR 27.7–87.5), p = 0.0003. Students in QCPR group performed significantly higher percentage of fully released chest compressions (71% [IQR 24.5–99.0] vs 24% [IQR 2.5–88.2]; p = 0.005) and better chest compression rate (117.5/min [IQR 106–123.5] vs 125/min [115–135.2]; p = 0.001). In secondary school students, a training for chest compressions based on a real-time feedback software (Laerdal QCPR^®^) guided by an instructor is superior to instructor-based feedback training in terms of chest compression technical skill acquisition.

**Trial Registration**: Australian New Zealand Clinical Trials Registry ACTRN12616000383460

## Introduction

High-quality chest compressions are pivotal to improve survival from cardiac arrest. A growing body of evidence highlights the association between quality of resuscitation manoeuvres and patients’ outcome. [1–6] Adequate rate, depth, recoil and hands position constitute what international guidelines [7,8] define high-quality chest compressions and when applied to cardiopulmonary resuscitation (CPR) they are associated with improved survival rates in the settings of both in-hospital and out-of-hospital cardiac arrest. Indeed, adequate rate and depth have been associated with better blood flow and oxygen delivery to the heart and brain and with an increased rate of return of spontaneous circulation (ROSC) and neurologically intact survival-to-hospital discharge. Incomplete chest recoil has been associated with reduced venous return to the heart and consequently reduced mean arterial pressure and coronary and cerebral perfusion pressures [5,9,10] Recently, a number of real-time automated feedback devices have been introduced in order to enhance both training and performance of CPR [11–13]. They generally provide a feedback based on direct assessment of current parameters of CPR and their real-time visualization through a software interface. Trainees may therefore read the current CPR parameters and eventually adopt corrective measure to promote a better agreement with guidelines recommendations. When applied in learning strategies involving different healthcare professionals and students, these devices improved CPR acquisition, retention and adherence to parameters recommended by guidelines. [14] There is a widespread recognition of the importance of including basic life support—defibrillation (BLS-D) manoeuvres in the school curriculum. This policy has been endorsed by different scientific societies including the European Resuscitation Council (ERC) along with governmental authorities. [15,16] Studies investigating BLS education among children and teenagers reported encouraging results. [17,18] However, the best method for CPR education has not been established. [15,19] Laerdal QCPR^®^ is a real-time feedback software able to measure CPR quality which can be connected wireless to a training mannequin (Laerdal Resusci Anne^®^). To our knowledge, no data are available about its use as part of CPR training among lay school students. The aim of this randomized trial was to assess the effectiveness of a training with Laerdal QCPR^®^ for the acquisition and retention of chest compression technical skill compared to a standard instructor-based training among secondary school students.

## Methods

We obtained the approval from the Ethics Committee of the University Hospital Paolo Giaccone, Palermo, Italy (Comitato Etico Palermo 1; approval number: 2/2016). We also obtained written informed consent from parents or legal representative of minor participants or directly from participants if over 18 years of age. We prospectively registered the trial in the Australian New Zealand Clinical Trials Registry (ACTRN 12616000383460). We performed this randomized study (allocation ratio 1:1) at the Secondary School “Liceo Scientifico Statale Stanislao Cannizzaro”, in Palermo, Italy. All study phases were performed from January to May 2016. The School Direction Committee approved the protocol of the study and provided information to students and their families before the trial starting date. We planned to exclude students who refused to join the trial for any reason. The trial consisted of three phases: 1) interactive frontal lesson on BLS-D and high quality chest compressions; randomization into two groups: QCPR group and Standard Feedback from instructor (SF) group; 2) training on chest compressions according the randomization group; 3) final evaluation.

### Phase 1

In the first phase, a trained instructor performed a 30-minute interactive frontal lesson about cardiac arrest and BLS-D to all participating students. The instructor demonstrated the BLS-D sequence, including recognition of cardiac arrest, call for help, early high-quality CPR. The instructor emphasised characteristics of high-quality chest-compressions, namely adequate compression rate, depth, recoil and hands position according to the European Resuscitation Council Guidelines 2015. [7] The instructor performing the interactive frontal lesson was blinded to group allocation of each student and he did not participate in the following trial phases. During this phase, an investigator, who did not join the frontal lesson, assigned an identification code to each present participant and performed the randomization. At the end of the lesson, participants were randomized in two parallel groups: 1. QCPR group and 2. SF group.

### Phase 2

At the beginning of the second phase, participants of both groups familiarised with the equipment/devices used for their training. Two instructors were involved in this phase, one for all students of each group. Students randomized in QCPR group received training by the instructor on chest compressions using a mannequin connected to a personal computer running specific software proving real-time electronic feedback about CPR quality. They were taught on the use of QCPR software, interpretation of real-time data and the parameters they should reach. Firstly, students of both groups started to practice until they reached a minimum sufficient technical skill to perform a 2-minute training session according to the instructor in charge. Secondly, the QCPR group performed a 2-minute training session taking into account the real-time data of the software trying to correct themselves to reach the high-quality targets. The SF group received feedback on their practice during the 2-minute training session according to instructor’s opinion. Thirdly, at the end of the session, all participants received a feedback on their performance: in the QCPR group the feedback was based on results from the software whereas in the SF group on the instructor’s judgment. Participants could repeat the 2-minute training session if they did not reach a certain level of ability, namely 60% of compression score in the QCPR group according to the software or a positive overall judgment from the instructor. Compression score is a balanced overall parameter of chest compression quality calculated by the software (see *Equipment and devices*). The value of 60% for compression score was decided by consensus among investigators as the acceptable minimum overall quality level of technical skill after training for students. This decision took into account information from manufacturer and investigators’ experience and personal data.

### Phase 3

**Fig 1:**
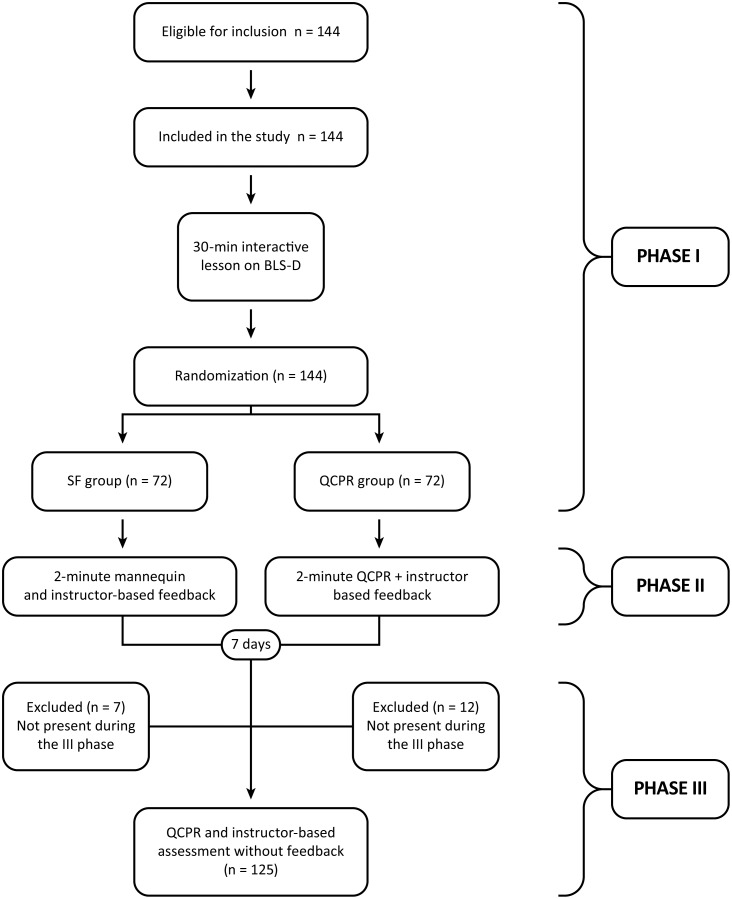
Flow diagram of the trial.

Seven days later, all the students who participated in the Phase 2 of the trial were asked to repeat a 2-minute chest compression session without feedback neither from the software nor from the instructor. All the sessions were analysed and registered with Resusci Annie Wireless SkillReporter^®^. An instructor, blinded to the randomization group, who did not participate in previous phases, judged students’ performance giving a dichotomous overall judgment. The instructor was not able to see real-time data from the software during the session. Students who did not attempt all the trial phases were excluded from the study. Instructors taking part in this trial did the standard training provided by the Italian Resuscitation Council. The flow diagram of the trial is reported in Fig 1.

### Equipment and devices

In this trial we used both standard mannequins for BLS-D training (Laerdal Resusci Anne^®^) and mannequins able to connect wireless with a software for real-time feedback and measurement of CPR quality (Laerdal Resuscie Anne^®^ QCPR^®^). The software used was Resusci Anne Wireless SkillReporter^®^, which is the personal computer version of Laerdal QCPR^®^. The software is able to measure all the relevant aspects of CPR. Since our study focused on chest compressions, we used the software in the *compression only* mode and considered only the available parameters related to chest compressions. The software was able to evaluate/measure hand position, compression rate, compression depth and chest release. For these parameters, we set the target measures according to European Resuscitation Council guidelines 2015. [7] The software is able to give real-time feedback on the performance proving a user-friendly graphics and colour code. At the end of a session, the software is also able to calculate an overall parameter, called compression score, which takes into account all the others, ranging from 0 to 100%. Moreover, single parameters are summarised in final overall values. More detailed information on software scoring could be retrieved in the manufacturer’s website (http://cdn.laerdal.com/downloads/f3943/Att_2_to_00021778.pdf). All the mannequins contained hook inside the chest needing a 30-kg strength to create a 5-cm depression.

### Sample size calculation and statistical analysis

We estimated that, with a sample size of 118, the study would have a 90% power to detect a minimum difference of 15% points in compression score at 7 days from the training between the two groups (with α = 5%). We expected a standard deviation (SD) of 25% points. This analysis came from a pilot study we performed one year before testing the Laerdal QCPR^®^ software for chest compressions acquisition in a similar population (unpublished data). The sample size was increased by 10% for non-parametric tests use (when appropriate). A further 10% was added for balancing drop out/lost of follow-up students, according to our experience. We analysed variables distribution with the D’Agostino-Pearson test and checked the plot distribution of the data for detection of skewness. Homoscedasticity was tested by the F-test. We calculated and reported mean and SD for variables with a normal distribution. In case of normality, a Student’s t-test was adopted. We expressed variables without a normal distribution as median and interquartile ranges (IQR, 25^th^– 75^th^) and comparisons were performed with Mann-Whitney U-test. Mann-Whitney U and the test statistic Z (corrected for ties) were reported as a measure for differences between groups. A frequency table was constructed and the chi-square test or Fisher’s exact test were used for comparisons of proportions. A two-tailed p < 0.05 was considered statistically significant. Statistical analysis was performed using MedCalc^®^ for Windows, version 9.5.0.0 (MedCalc^®^ Software, Mariakerke, Belgium). We adopted the Consolidated Standards of Reporting Trials (CONSORT) guidelines for this trial (see S1 Appendix). [20]

### Randomization

The selection of classes participating in the trial was done by randomization with sealed envelopes. All classes at fourth and fifth year of study course were available for the enrolment according to School Direction decision. An appropriate number of classes were enrolled to match the sample size considering a mean value of 20 students per class. At the end of the lesson in phase 1, after assigning unique identification codes to participants, an investigator generated the randomization list using an on-line, random sequence generator. The randomization was performed in block of 4, 6 and 8.

### Outcomes and data

The primary outcome of the study was the compression score calculated by Laerdal QCPR^®^ software at 7 days from the training. The secondary outcomes derived from the software were: 1) compression rate 2) compression depth 3) percentage of compressions with complete chest release 4) percentage of compressions with correct hand position. Other secondary outcomes were: 5) overall dichotomous judgment from the instructor who judged students’ performance (adequate/not adequate); 6) proportion of students reaching a compression score of 75% (minimum score for an “Advanced CPR Performer” according to manufacturer’s indications and confirmed by consensus among authors) and 90%; 7) proportion of students reaching a compression score of 90%; 8) 1–5 Likert-scale score from participants investigating the degree of their comfort and how they enjoyed the overall experience. We collected data on students’ sex, age, weight, height and any prior CPR hands-on training.

## Results

We randomized a total of 144 participants, 72 in QCPR group, 72 in SF group. All students completed the training phase (Phase 1 + Phase 2). Five students in the QCPR group (6.94%) did not reach the minimum compression score of 60% after the 2-minute training session and repeated it. After a second 2-minute training, all 5 students reached the minimum compression score of 60%. Median overall compression score of QCPR group at the end of training was 96 (IQR 86.7–98.0). Six students (8.33%) in the SF group did not reach the minimum overall quality as judged by the instructor. Students repeated the 2-minute training session after instructor-based feedback and all reached the minimum overall quality. Twelve students in QCPR group and 7 students in SF group were not present during the third phase of the trial and were therefore excluded. A total of 125 students completed all study phases, 60 in QCPR group and 65 in SF group. Characteristics of students who completed all study phases are summarised in Table 1.

**Table 1:** Characteristics of students who completed all study phases.

	QCPR group (n = 60)	SF group (n = 65)	Overall (n = 125)	P—value
**Sex**	M = 46; F = 14	M = 39; F = 26	M = 85; F = 40	P = 0.071
**Age, years (Median, IQR)**	17.0 (17.0–18.0)	17.0 (17.0–18.0)	17.0 (17.0–18.0)	P = 0.9526
**Weight, Kg (Median, IQR)**	63.0 (58.5–70.0)	62.0 (55.0–71.2)	63.0 (57.7–70.0)	P = 0.869
**Height, cm (Median, IQR)**	174.5 (167.5–180)	173 (166.7–178.0)	174 (167–180)	P = 0.337
**Prior experience with hands-on CPR training**	3	2	5	P = 0.670

**Fig 2:**
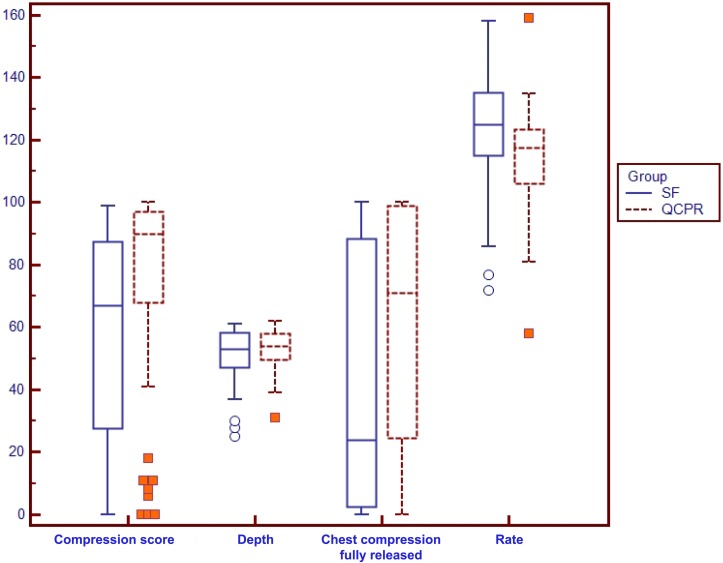
Box plots graph showing 1) compression score expressed as percentage 2) depth of chest compressions in millimeters 3) percentage of compressions with full release of the chest 4) rate of chest compressions (compressions/minute).

A significantly higher compression score was observed in students in QCPR group (median 90, IQR 81.9–96.0) compared to SF group (median 67, IQR 27.7–87.5), p = 0.0003, U = 1218, Z = 3,619 (Fig 2). Furthermore, we observed a statistically significant difference in proportion of students reaching a compression score ≥ 75% among QCPR group (42/60 = 70.0%) compared to SF group (26/65 = 40.0%), p = 0.001 corresponding to the lower limit of advanced CPR performer’s score range according to manufacturer’s instructions. We also observed a statistically significant difference between the two groups when we considered a compression score ≥ 90%, QCPR group 32/60 = 53.3% vs SF group 13/65 = 20.0%, p = 0.0002. A statistically significant difference between the two groups was observed for the rate of chest compressions, with a median chest compression rate of 117.5/min (IQR 106–123.5) in QCPR group and 125.0/min (IQR 115–135.2) in the SF group, p = 0.001, U = 1284, Z = 3.292. Considering compression depth, we did not observe a statistically significant difference between QCPR group (median 54.0 mm, IQR 49.5–58.0) and SF group (53.0 mm, IQR 47.0–58.2), p = 0.58, U = 1839, Z = 0.547. We observed a statistically significant difference between the two groups for the percentage of chest compressions with complete release, with a median of 71% (IQR 24.5–99.0) in QCPR group and 24% in SF group (IQR 2.5–88.2), p = 0.005, U = 1392, Z = 2.762 (Fig 2). We did not observe a statistically significant difference between the two groups for the percentage of chest compressions with correct hands position (p = 0.054, U = 1744, Z = 1.921). When we considered the instructor-based dichotomous overall judgment, we did not observe a statistically significant difference between proportion of students performing inadequate chest compressions in QCPR group (8/60 = 13.3%) and SF group (10/65 = 15.4%) p = 0.943. Students among groups did not answer differently to a 1–5 Likert questionnaire investigating degree of comfort (QCPR group, median 4.0, IQR 4.0–5.0; SF group, median 4.0, IQR 4.0–4.25, p = 0.22) and how they enjoyed the overall experience (QCPR group, median 3.0, IQR 3.0–4.0; SF group median 3.0, IQR 3.0–4.0).

## Discussion

In this randomized trial, secondary school students participating to a training on chest compressions with an instructor and a real-time electronic feedback system (Laerdal QCPR^®^ in form of Laerdal Resusci Anne Wireless SkillReporter^®^) showed a higher technical skill acquisition compared to training with standard instructor-based feedback only. The primary outcome of the study was an overall measure of chest compression quality provided by the software. Although the gold-standard for the certification of the CPR technical skill acquisition is the instructor’s judgment, we decided to use compression score as primary outcome since it provided a quantitative and sensitive assessment of parameters corresponding to high-quality chest compressions, namely compression rate, depth, recoil and hand position during its performance. When considering chest compression parameters separately, students in QCPR group showed better chest recoil and compression rate. We did not observe difference in depth and hand position. We may argue that the observed difference in compression score should be attributed mainly in the difference in chest recoil and, to a minor extent, in compression rate. Chest recoil may be difficult to be taught and recognised during chest compressions performance. Our data suggest that feedback from the software may improve the acquisition of the ability to perform chest compressions with adequate recoil.

Although our trial investigated the effect of Laerdal QCPR^®^ in secondary school students, it should be noted that participants followed a training program consisting on an interactive frontal lesson led by instructors who also led the familiarization with the devices, helped the interpretation of real-time results and discussed with each student the final software report during the training phase. We cannot exclude a different effect with the use of the same feedback system without the support of an instructor since the effect of the instructor and feedback system could not be disentangled due the specific trial design. Notably, we decided to evaluate the chest compressions performance at 7 days in order to avoid the previously reported *boosting effect*, which may occur soon after an educational intervention, leading to dilution of the effect. [21,22]

Real-time feedback systems are listed by guidelines among technological aids potentially useful for education and implementation of CPR. [23] A number of feedback devices have been developed, ranging in complexity from metronomes providing real-time information of chest compression rate during training or actual resuscitation to more sophisticated devices which provide a feedback on all parameters of high-quality CPR and report a final score resulting from a balanced analysis of each parameter and its deviation from a pre-set value. A systematic review of studies investigating the usefulness of CPR feedback/prompt device during training and CPR performance supported the use of these devices in order to improve CPR skill acquisition and retention. [14] Another systematic review and meta-analysis investigated the use of audiovisual feedback devices by healthcare professionals both in real-life CPR settings and in mannequin-based scenarios. [24] In both real-life and simulated scenarios, these devices improved adherence to guidelines recommendation of resuscitation attempts. However, there is not sufficient evidence to support the use of these devices to improve patients’ outcomes. [24–27] Recently, an observational study investigated Laerdal Resusci Anne Wireless SkillReporter^®^ for training of lay people in chest compression only CPR. [28] After a 5-minute instructor-based training on CPR with an emphasis of which parameters constitutes high-quality chest compressions, participants were invited to perform a 2-minute session of hands-only CPR. Notably in this study performance of 81 lay people without prior experience of CPR training was compared to that of a group of 74 healthcare professionals. Investigators did not observe a statistically significant difference of chest compressions performance between lay participants and healthcare professionals when they considered rate, depth, chest wall recoil and hand position, supporting the feasibility and efficacy of a short training of hands-only CPR supported by real-time feedback involving lay people. To our knowledge, our study is the first randomized trial investigating the effect of Laerdal QCPR^®^ for chest compression training in secondary school students.

In our trial, the proportion of students performing inadequate chest compressions according to the instructor’s overall dichotomous judgement during the assessment was not significantly different between groups. The instructor judged students’ performance evaluating chest compressions characteristics globally for the whole 2-minute period. It may be hypothesized that the software may be more sensible than instructor to see minor deviation from high quality characteristics of chest compressions and/or more able to balance adequateness of the manoeuvres in relation to time.

Our study has limitations. Firstly, we assessed the effect of our intervention only at 7 days without further follow-up. We cannot exclude different effect size of intervention at a longer follow-up. Another limitation may be the 2-minute length of training in both groups. It may be argued that longer training sessions would have led to a different effect. We decided to adopt a 2-minute training session in order to complete the second trial phase in a reasonable timeframe within the same day for all students involving the same instructor for each group. However, the training duration was identical in both groups and we did not observe a statistically significant difference in the proportion of students needing further training in both groups. The training session of the SF group was performed by an instructor who was not blind to the group assignment and this may potentially introduce a bias. Due to the design of the trial, this was unavoidable. However, the instructor was an official Italian Resuscitation Council instructor whose responsibility consists also in recognizing the effectiveness of the manoeuvres in a standardized fashion. We aimed to recreate the standard training usually used for students to have a “real-setting” comparison to the QCPR group.

Finally, we investigated the use of a real-time feedback for chest compression only not including other CPR manoeuvres (e.g. ventilation). It should be noted that guidelines highlight the option of chest compression only CPR for lay people since this may increase the willingness to perform CPR by bystanders and early institution of high-quality chest compressions may represent the single intervention with a major role in the overall patient outcome. [7,23]

## Conclusions

In secondary school students, a training for chest compressions based on the use of a real-time feedback software (Laerdal QCPR^®^) guided by an instructor is superior to instructor-based feedback training in terms of overall chest compression technical skill acquisition. The effect may be attributed to a difference in the ability to perform adequate chest recoil and compression rate.

## Supporting Information

S1 Appendix CONSORT Checklist. (PDF)

S2 Appendix Dataset of the study. (XLSX)

S3 Appendix Study protocol approved by the Ethics Committee. (PDF)
