# Supplementary material for: Use of a Real-Time Training Software (Laerdal QCPR®) Compared to Instructor-Based Feedback for High-Quality Chest Compressions Acquisition in Secondary School Students: A Randomized Trial
Source: PLoS One. 2017 Jan 5;12(1):e0169591. doi: 10.1371/journal.pone.0169591 (PMC5215847; doi:10.1371/journal.pone.0169591)
Supplement: S3 Appendix — (PDF) [file pone.0169591.s003.pdf]

UNIVERSITÀ DEGLI STUDI DI PALERMO

DIPARTIMENTO DI BIOPATOLOGIA E BIOTECNOLOGIE MEDICHE

Sezione Anestesia Analgesia Rianimazione e Terapia Intensiva:
Responsabile : Prof. Antonino Giarratano
e-mail: antonino.giarratano@unipa.it

dibimed

USE OF A REAL-TIME TRAINING SOFTWARE (LAERDAL QCPR®) COMPARED TO INSTRUCTOR-BASED FEEDBACK FOR HIGH-QUALITY CHEST COMPRESSIONS ACQUISITION IN SECONDARY SCHOOL STUDENTS: A RANDOMIZED TRIAL

Study Protocol

Rationale

Cardiac arrest s a leading cause of death worldwide. Correct maneuvers of Basic Life Suport – Defibrillation (BLS-D) are pivotal to improve victims' outcome. The oxygen debt of vital organs leads to irreversible damage after 4-6 minutes without blood flow. Rapid onset of the maneuvers and high quality BLS-D may help to prevent the onset of these damages. Chest compressions are one the most import maneuver for the improvement of the outcome. The European Resuscitation Council Guidelines 2015 underline the importance of cardiopulmonary resuscitation performed by bystanders while waiting for the arrival of medical support (1-2). The education is an essential part of lay people and medical training on cardiopulmonary resuscitation. The education on cardiopulmonary resuscitation of primary and secondary school students is an international priority. The role of high quality chest compressions is increasingly recognized as pivotal for the outcome of cardiopulmonary resuscitation and for the reduction of organs damage. The gold standard for BLS-D training if the role of a qualified instructor. However, new electronic tools for feedback during the training give trainees information on their performance (3-4). Laerdal QCPR is a computerized system connected to a manikin for BLS-D training able to give real-time feedback on chest compressions quality. Evaluated parameters encompass: compression rate, depth, chest recoil, and hand position. (5). The system is able to calculate an overall score defined as compression score, which summarize all these parameters.

To our knowledge, a comparison between instructor-based training and software-based training

(Laerdal QCPR) in secondary school students in a randomized trial has never been performed.

Aim of the study

This study aims to evaluate the efficacy of feedback by a computerized skill-reporting system (Laerdal QCPR) during the cardiopulmonary resuscitation training among secondary school students. The study focuses on chest compression training. The efficacy of feedback from Laedarl QCPR will be compared to the gold standard that is feedback from a qualified instructor.

Study design

Randomized trial. The study will include secondary school students of the last two years of study course. They will follow a frontal lesson performed by a qualified instructor about cardiac arrest, BLS-D and high-quality chest compressions. Students will be randomized in two groups 1) QCPR group 2) control group. In the QCPR group students will familiarize with equipment (manikin and QCPR software). Then, they will perform a 2-minute training session guided by the feedback from the software evaluating compression rate, depth, chest recoil and hand position. After this session, students will have a discussion with the instructor basing on the output of the system. Students will proceed to the next phase of the trial if they reach a compression score >=60% otherwise they will repeat the training session until they reach this cut-off. In the other group, students will performed a 2-minute training session guided by the instructor and at the end, they will discuss with him about their performance. Students of this group will proceed to the next phase according to instructor's judgment. After 7 days, all students will perform a 2-minute session of chest compressions on the manikin connected to the QCPR system for the evaluation. An instructor will also evaluate the session. Neither the student nor the instructor will be able to see the output of the QCPR software during or after the session. Students will also asked to complete an appreciation subjective questionnaire. We will perform the study at the Liceo Scientifico Statale S. Cannizzaro. The primary outcome of the study if the compression score which is an overall score of chest compression quality calculated by the

software.

Inclusion criteria

Students of the last two years of study course of the secondary school Liceo Scientifico Statale S. Cannizzaro

Exclusion Criteria

Students not present at one the study phases
Students who refuse to participate. Students who do not give informed consent.

Materials

To perform all trial phases we will use one manikin connected to the QCPR system and a personal computer running the specific software (Resusci Annie Skill Reporter – QCPR – laerdal) and one simple training manikin (Resusci Annie). The materials for this trial belong to the Department of Biopathology and Medical Biotechnologies (DIBIMED), Section of Anesthesia, Analgesia, Intensive Care and Emergency, University of Palermo and Laerdal Italy.

References

1. Koenraad G. Monsieursa,b, Jerry P. Nolanc, Leo L. Bossaerte, Robert Greiff,g,Ian K. Maconochieh, Nikolaos I. Nikolaoui, Gavin D. Perkinsj,p, Jasmeet Soark,Anatolij Truhlářrl,m, Jonathan Wyllien, David A. Zidemano,on behalf of the ERC Guidelines 2015 Writing Group. European Resuscitation Council Guidelines for Resuscitation 2015. Resuscitation 95 (2015) 1–80.

2. Sasson C1, Rogers MA, Dahl J, Kellermann AL. Predictors of Survival From Out-of-Hospital Cardiac Arrest: A Systematic Review and Meta-Analysis . Circ Cardiovasc Qual Outcomes. 2010 Jan;3(1):63-81

3. Beck S, Issleib M, Daubmann A, Zollner C. Peer education for bls-training in schools? Results of a randomized-controlled, noninferiority trial . Resuscitation. 2015 Sep;94:85-90

4. Lynn P. Roppoloa, Rahm Heymannb, Paul Pepea, James Wagnerc,, Bradford Commonsa,Ronna Millera, Emilie Allend, Leyla Hornee, Michael P. Wainscotta, Ahamed H. Idris . A randomized controlled trial comparing traditional training in cardiopulmonary resuscitation (CPR) to self-directed CPR learning in first year medical students: The two-person CPR study. Resuscitation 82 (2011) 319–325

5.http://cdn.laerdal.com/downloads/f2729/Scoring_CPR_November_v2.pdf

Protocollo di studio

Uso di un sistema di Feedback elettronico associate a manichino di adestramento per l'approvazione delle manovre di massaggio cardiaco di qualità tra studenti della scuola media superiore. Studio randomizzato.

Trial ID: ACTRN12616000383460

Razionale dello studio

L'arresto cardiocircolatorio è una delle principali cause di morte nel mondo. Fondamentale per un'eventuale outcome positivo della vittima di arresto cardiocircolatori è l'esecuzione delle corrette manovre di Basic Life Support Defibrillation – Supporto vitale avanzato (BLSD). Il deficit di ossigenazione degli organi vitali può condurre a lesioni irreversibili già dopo 4-6 minuti di assenza di circolo. Le possibilità di prevenire questi danni dipendono dalla rapidità e dall'efficacia delle manovre di primo soccorso, in primo luogo le compressioni toraciche poiché rappresentano uno degli elementi principali per migliorare la sopravvivenza. Gli orientamenti ERC 2015 sottolineano l'importanza delle rianimazione praticata dagli astanti in attesa dei soccorsi sanitari e del coordinamento tra chi inizia le manovre BLS-D e il primo soccorso medico in termini di prognosi e sopravvivenza. L'educazione,perciò, è una parte essenziale della formazione del personale laico e non ed il training può essere uno strumento efficace per la formazione di competenze di base (1-2). La formazione sulla rianimazione cardiopolmonare degli studenti delle scuole medie superiori ed inferiori è una priorità internazionale. Si è sempre più enfatizzato l'importanza

delle compressioni toraciche di alta qualità per il buon esito delle procedure rianimatorie e per ridurre la velocità di deterioramento degli organi vitali. Nonostante ad oggi il gold standard per l'addestramento alle manovre BLS-D sia l'istruttore qualificato, i nuovi dispositivi di feedback offrono agli allievi chiare indicazioni su come migliorare le prestazioni della RCP e le competenze acquisite.

Il Laerdal QCPR è un sistema computerizzato collegato ad un manichino per l'addestramento al BLS-D in grado di fornire, attraverso un'interfaccia grafica, un feedback in tempo reale sulla qualità delle compressioni toraciche. I parametri presi in considerazione dal software sono: frequenza e profonditá delle compressioni, numero di compressioni per ciclo, retrazione toracica dopo ogni compressione e posizione delle mani (5). Il sistema è capace di calcolare un punteggio che prende in considerazione tutte qusti parametri delle compressioni toraciche definito come compression score (5). Non è mai stato testato il confronto tra un training mediante istruttore ed un training guidato da un software computerizzato (Laerdal QCPR) in un trial randomizzato riguardante studenti si scuola media superiore (3-4).

Scopo dello studio

Il presente studio si propone di valutare l'efficacia di un feedback in tempo reale tramite il sistema computerized skill-reporting system (QCPR) durante l'addestramento di studenti di scuola media superiore ai fini di migliorare l'apprendimento e il mantenimento delle competenze in rianimazione cardiopolmonare (RCP), nello specifico delle compressioni toraciche, e soprattutto ottenere prestazioni migliori in situazione reale. Tale feedback verrà confrontato con il gold standard per il training che è il feedback da parte di un'istruttore certificato.

Disegno dello studio

Studio randomizzato. Lo studio verrà condotto con gli studenti frequentanti gli ultimi due anni di una scuola media superiore che verranno sottoposti ,in una prima fase, ad una lezione frontali svolte da un istruttore qualificato, riguardanti l'arresto cardiaco, il BLSD e le compressioni toraciche di alta qualità. Gli studenti saranno successivamente randomizzati in due gruppi: 1) gruppo QCPR 2) gruppo controllo. Nel gruppo QCPR gli studenti verranno sottoposti ad un periodo di familiarizzazione con il manichino ed il software del QCPR, successivamente effettueranno un ciclo di due minuti di compressioni toraciche guidati dal software che valuterà la frequenza e la profondità delle compressioni , il recoil della gabbia toracica e la posizione delle mani. A questo seguirà una discussione con l'istruttore sulla loro performance basata sulle informazioni date dal software. Gli studenti potranno passare alla fase successiva se avranno ottenuto un punteggio compression score (calcolato dal software) >=60%. In caso contrario gli studenti effettueranno un nuovo ciclo di training. Nel gruppo controllo gli studenti eseguiranno un ciclo di due minuti di compressioni guidati dall'istruttore, al termine del quale, dopo un breve debrifing, eseguiranno un nuovo ciclo di compressioni finchè l'istruttore qualificato giudicherà raggiunti i criteri di compressione di alta qualità. Dopo 7 giorni tutti gli studenti verranno valutati tramite l'esecuzione di un ciclo di compressioni toraciche con manichino e software QCPR per la valutazione. Anche un instruttore valuterà la prova dello studente. La valutazione finale verrà eseguita senza che né lo studente né l'istruttore potrà vedere i risultati descritti dal software durante e dopo la prova. Verranno sottoposti ad un questionario che valuterà il loro livello di soddisfazione. La sede dello svolgimento dello studio è il Liceo Scientifico Statale S. Cannizzaro. Il primary outcome dello studio è il compression score, un overall score calcolato dal software che prende in considerazione le caratteristiche delle compressioni toraciche.

Criteri di inclusione

UNIVERSITÀ DEGLI STUDI DI PALERMO
DIPARTIMENTO DI BIOPATOLOGIA E BIOTECNOLOGIE MEDICHE
Sezione Anestesia Analgesia Rianimazione e Terapia Intensiva:
Responsabile : Prof. Antonino Giarratano
e-mail: antonino.giarratano@unipa.it
dibimed

Studenti 4 e 5 anno della scuola media superiore Liceo Scientifico Statale S. Cannizzaro.

Criteri di esclusione

Studenti assenti ad almeno un giorno di formazione.
Studenti che rifiutano la partecipazione allo studio e che non danno consenso informato.

Materiali

Al fine di portare a termine il trial, si prevede di utilizzare per entrambe le giornate descritte nei precedenti paragrafi un manichino simulatore connesso ad personal computer dove funzionerà il software specifico (Resusci Annie Skill Reporter – QCPR – Laerdal) ed un manichino semplice (Resuscie Annie). I materiali impiegati per lo svolgimento di questo trial appartengono a Dipartimento di Biotecnologia e Biotecnologie Mediche, Sezione di Anestesia Analgesia Rianimazione e Terapia Intensiva, alla Scuola di Medicina e Chirurgia dell'Università degli Studi di Palermo e a Laerdal Italia.

Bibliografia

1. Koenraad G. Monsieursa,b, Jerry P. Nolanc, Leo L. Bossaerte, Robert Greiff,g,Ian K. Maconochieh, Nikolaos I. Nikolaoui, Gavin D. Perkinsj,p, Jasmeet Soark,Anatolij Truhláˇrl,m, Jonathan Wyllien, David A. Zidemano,on behalf of the ERC Guidelines 2015 Writing Group. European Resuscitation Council Guidelines for Resuscitation 2015. Resuscitation 95 (2015) 1–80.

2. Sasson C1, Rogers MA, Dahl J, Kellermann AL. Predictors of Survival From Out-of-Hospital Cardiac Arrest: A Systematic Review and Meta-Analysis . Circ Cardiovasc Qual Outcomes. 2010 Jan;3(1):63-81

3. Beck S, Issleib M, Daubmann A, Zollner C. Peer education for bls-training in schools? Results of a randomized-controlled, noninferiority trial . Resuscitation. 2015 Sep;94:85-90

4. Lynn P. Roppoloa, Rahm Heymannb, Paul Pepea, James Wagnerc,, Bradford Commonsa,Ronna Millera, Emilie Allend, Leyla Hornee, Michael P. Wainscotta, Ahamed H. Idris . A randomized controlled trial comparing traditional training in cardiopulmonary resuscitation (CPR) to self-directed CPR learning in first year medical students: The two-person CPR study. Resuscitation 82 (2011) 319–325

5.http://cdn.laerdal.com/downloads/f2729/Scoring_CPR_November_v2.pdf
